# Kawasaki disease: a confusing trigger in hemophagocytic lymphohystiocitosis

**DOI:** 10.1186/1939-4551-8-S1-A49

**Published:** 2015-04-08

**Authors:** Mariana Forti, Briza Botelho, Mayra Dorna, Claudia Ivo, Pedro Sakane, Katia Kozu, Antonio Carlos Pastorino, Ana Paula Moschione Castro, Cristina Jacob

**Affiliations:** 1 Unit of Allergy and Immunology, Brazil; 2 Unit of Infectious Diseases, Brazil; 3 Unit of Rheumatology Diseases, Brazil

## Background

Hemophagocytic Lymphohystiocitosis (HLH) is a severe hyper inflammatory condition that demands prompt recognition and aggressive treatment. It can be triggered by different conditions including other inflammatory diseases like Kawasaki disease (KD). The diagnosis of both may be difficult and their symptoms can overlap.

## Methods

Case reporting.

## Results

A 4 year-old boy was hospitalized to investigate a fever of unknown origin. At day 24 of persistent fever, he presented rash and left coronary artery dilatation (3.6mm) were detected, and was diagnosed with atypical KD. He received high-dose immunoglobulin (IVIG, 2g/Kg, in 3 days) but fever had persisted and coronary dilatation worsened (left coronary artery 4.2mm and anterior descending artery 2.8mm and circumflex artery 2.6mm). At day 33 he presented severe clinical deterioration and fulfilled diagnostic criteria for HLH. As KD was considered the HLH trigger, a modified protocol for treatment was applied. He was treated with IVIG and dexamethasone (initial dose 10mg/m²/day) with remission of the fever after the first dose and progressive clinical and laboratorial improvement. Due to the persistence of splenomegaly, thrombocytopenia and neutropenia, he received one dose of etoposide at day 48 with resolution of the cytopenias. Clinical and laboratory parameters improved to normal levels, coronary arteries dilatation reduced (left coronary artery 2.2mm, anterior descending artery 2.0 mm and circumflex artery 1.6mm), allowing progressive reduction of the corticosteroid. Splenomegaly is the only abnormality that still persists after 40 days of treatment.

## Conclusions

The case showed that HLH may be a complication of KD and that the adequate treatment for HLH, even with less aggressive immunossupression, allowed fast and impressive clinical improvement. It is important to be aware of this possible association to guarantee a better long term prognosis.

## Consent

Written informed consent was obtained from the patient for publication of this abstract and any accompanying images. A copy of the written consent is available for review by the Editor of this journal.

